# Physiologie des Temperamens ou Constitutions

**Published:** 1830-07-01

**Authors:** 


### Medico-Chirurgical Review,

No. XXV.

APRIL 1 to JULY 1, 1830.

### I.

### ON TEMPERAMENT.

Physiologie des Temperamens ou Constitutions; nouvelle Doctrine applicable a la Médécine-pratique, &c. Par *F. Thomas*, (de troisième) D.M.P.: à Paris.

[Prize-Review.]

If great diversity of sentiment indicate great difficulty in the subject investigated, there are few points of a more abstruse and perplexing character than the theory of those physical varieties which obtain in different individuals of the human species. That a mechanism essentially the same wherever met with—in whatever quarter of the globe and among whatever people—could furnish such endless modifications of internal function and of external feature, was not to have been expected. Every face wears a physiognomy characteristically its own; every mind presents qualities peculiar to itself. No similarity of circumstance can secure similarity of character. Individuals, born of the same parents and in the same house, suckled on the same breast and nursed on the same knee, educated by the same masters and subjected to the same authority, introduced to the world at the same period and moving in the same society—are different, frequently as different as the clumsy Japanese and the elegant Circassian are in body, or the ferocious Tartar and the harmless Hottentot in mind. Though all are generically alike, all are specifically dissimilar; though all are cast into the same mould, all come out with distinguishing personalities. Beauty adorns one, deformity marks another; judgment, or imagination forms the prominent quality of a third. Indolence and activity, talent and stupidity, passions the most headstrong and affections the most engaging, may thus be found growing out of the same soil, associating under the same roof, and peculiarizing individuals born and nurtured under the influence of the same circumstances.

How this is, and why this should be, are questions which have been more frequently asked than answered. If great differences appear between people separated from each other by great distances, the dissimilarity may not be inexplicable. If the Laplander, who is cradled in the storm, be unlike the Negro of Guinea, who is nursed in the sun-beam, it may not be deemed marvellous. Local peculiarities can accomplish, and have effected much; and, although the cave of Trophonius lays claim to more miracles than our faith can ascribe to it, the demonstration were not difficult which proved that variety of climate, of custom and of education has wrought mental and physical transformations, which some have rashly referred to original varieties of species. It is, however, vain and preposterous to stretch the agency of these modifying causes beyond a certain limit. To a certain limit their agency is undeniable; but those physiologists, who maintain that mere difference of external condition can explain every difference of corporeal and metaphysical character, betray sad embarrassment in many of their arguments.

When these constitutional peculiarities are understood in a general sense, they are denominated TEMPERAMENTS; when intended for individual application, they are styled IDIOSYNCRACIES. Temperament is the *genus*, idiosyncracy the *species*; but since each individual is marked by some distinguishing quality, through which he is made to differ from every other, there must be as many temperaments as men, the history of constitutional peculiarities would be the history of every member of the human race, and, therefore, minuteness on a subject of such extent were as vain as it would be useless. Leading points of dissimilarity alone can be considered, as they only can furnish general results, and the tender pencilling of shadowy individualities must be left for the study of the Novelist, whose aim is less instruction than amusement.

The ancients ascribed much more importance than we do to the knowledge of temperaments; and in their treatment of disease generally followed them as their guide to the choice of remedies. Corresponding to their four elements,—fire, air, earth, and water, and to the four qualities ascribed to these,—heat, cold, dryness, and moisture, four varieties of constitution were discovered and described. The blood was viewed by many of them as the first vital principle, if not the life, to which every constitutional peculiarity was referred; and as it departed *en masse* or in its separate elements from what was supposed to be a state of health, the system was inferred to suffer in proportion. If its red particles abounded, the constitutional habit was *sanguine;* if the phlegm were in excess, the person was *phlegmatic;* and as the yellow, or the black bile preponderated, the *choleric* or *melancholic* φυσις prevailed. These four κρασιες of Hippocrates Galen multiplied to nine; but this augmentation seems to have been little better than a useless refinement, for, except the last or *temperamentum ad pondus*, those which were added can scarcely be regarded in any other light, than as new appellations, or mere explanations of the first. Cullen has with his usual ingenuity examined how far the constitution of the blood may modify that of the system, and after a minute consideration of the subject concludes that "with respect to its aggregation, or with respect to the state and proportion of the several parts which compose it as an aggregate, it seems not only to be uncertain how far these circumstances give a difference of temperament, but, on the contrary, it seems probable that they never do so in any considerable degree."

While the humoral pathology reigned this theory of the ancients reigned along with it; and Stahl has with much address traced the connexion between the powers of mind and body in health and disease and the constitutional states of the animal fluids. When the system was phlegmatic the fluids were thin and unstimulating; when melancholic they were thick and inactive; when sanguine they were hot; when choleric they were acrid.

To assert that there is no truth in all this would be to say more than we are warranted, but it were not difficult to prove that it is not all true. If difference of constitutional temperament depend upon constitutional differences in the blood, it is natural to suppose that when a certain condition of this fluid obtains we should find a corresponding state of temperament; that when the blood appears thin and poor, the habit should be phlegmatic; when thick and rich, melancholic. The inverse of these propositions should also be true; when the habit is phlegmatic, the blood should be thin and poor; when melancholic, it should be richer and increased in consistency. But neither is the first proposition, nor its inverse correct; nor is it true that the average temperature of phlegmatic habits is lower than that of such as are choleric.

Dissatisfied with the *humoral* doctrine of Hippocrates and Galen, and the *humoro-mechanical* theory of Stahl, Haller attempted a new arrangement, grounded on irritability of the solids. When they united great firmness to great irritability, the habit was bilious; when they were firm, but less irritable, sanguine; when lax, but more irritable, melancholic; and when below par both in tonicity and irritability, the phlegmatic temperament prevailed. *Niederhuber* adopted a still simpler system, and ascribed every variety of constitution, not to corporeal difference, but to simple modifications of the vital power. *Cabanis* profited by all the doctrines which preceded him, and combined into one system, the humoral, mechanical, and vital theories. This physiologist makes six temperaments. The first four are those of Hippocrates, which he ascribes to the tone of the solids,—to the quantity and quality of the fluids,—to their proportion,—to the size and power of the lungs, heart, liver, and genital organs,—and to the sympathetic communications existing between them. The last two temperaments are the *muscular* and *nervous*, which result from the reciprocal predominancy of these systems, the one above the other. The views of *Richerand* are very similar to those of Cabanis, and his number of temperaments is the same. *Hallé* distinguishes them into *partial* and *general*. The *general* are eight. The first four, which are those of Hippocrates, he traces to the proportions maintained between the lymphatic and sanguineous systems. When they were balanced the result was the sanguine temperament; when the lymphatic system was in excess, the constitution was *pituitous;* and where the sanguineous exceeded, the person was melancholic. The fifth and sixth, or *plethorico-sanguine*, and *plethorico-lymphatic* habits, are founded upon the predominancy of those two systems from which these habits derive their name; and the seventh and eighth are the same as the fifth and sixth of Cabanis. The *partial* temperaments are divided into two classes, and arise out of visceral plethora, whether sanguine or lymphatic, and the energy of individual organs. Kruger, Metzler, Rosanstein, Schrocæder, Lawœz, Schmidt, and many others have written upon this subject, and, as may be expected, have variously modified the doctrines and divisions of those who went before them; but it is unnecessary to pursue this history further, to prove that little of importance has been added to the views of the ancients. The four cardinal points of Hippocrates have been advocated throughout, although sometimes under new designations, and his followers have rather been loading the points of the compass, which were already known, with a new nomenclature, than actually adding to their number. Yet one cannot easily discover wherein lies the strength of many of the arguments by which some of his temperaments have been defended. The nomenclature, at least, is certainly indefensible, for the melancholic have no more *atrabilis*, nor the sanguine more *blood*, than their neighbours; and a system so limited as the lymphatic, can scarcely be deemed important enough to constitute the temperament known under that name.

In introducing the author, whose work is now before us, it is not quite certain whether we shall be adding much to our stock of knowledge on the subject of Temperaments. His system, although advanced as new, only requires explanation to divest it of every claim to originality. It is adorned, no doubt, with a few novelties, and the charms of phrenological mysticism have been tastefully thrown over it; but its basis was long since laid, and part of its superstructure has been erected for ages. But, like all doctrines which are formed upon popular sentiments, and which, by interweaving their interests with those of leading national feelings, embark their claims to public favour upon the same bottom, the doctrine of Thomas has gained, and is now gaining on society, and if the signs of the times can be any guide, it would not exceedingly amaze us, were it eventually installed into the canons of our own College. His theory sets out with the position, that *the relative size of an organ indicates the relative energy of its functions; or, in other words, that the volume and functions of a part are relatively proportional.* Living bodies, he argues, are composed of matter disposed in organs, the nature and number of whose functions are generally regulated by the peculiaries of their structure. Each organ exerts a specific action which is peculiar to itself, and a reciprocal action which respects the system as a whole. All the organs of which the whole is composed, having thus a mutual dependence on each other accompanied with a distinct existence, one cannot exert more than its intended allotment of activity, without imparting a peculiarity of action to the whole. The most important organs are those which are contained in the three great cavities. Such as lie without are subservient; instruments by which these internal organs are assisted in discharging their own functions. In the first cavity lies the brain, which is the exclusive organ of intelligence and passion; in the second are placed the lungs and heart, which are the organs of sanguification and circulation; and within the abdomen are found the principal secreting organs, and such as are destined for the formation of chyle. Now, proceeds Dr. Thomas, it is found that in all animals the functions of these organs bear a constant relation to their size. When the brain is large, in proportion to the rest of the body, cerebral function predominates; when the viscera of the thorax exceed, the respiratory and circulating functions prevail; and when those of the abdomen, abdominal functions preponderate. In *Reptiles*, and other cold-blooded animals, the lungs are clumsy and unfit for active function. In the *Frog*, *Lizard*, and *Salamander*, they are mere sacs, internally divided into a few cells, and the heart is equally simple in construction; whereas in *Birds*, and *Mammalia*, in which both these organs are complicated, these functions are energetically performed. Among *Zoophytes* the abdomen constitutes nearly the entire animal; in *Insects* the mechanism is more complicated; among reptiles the thoracic, or cerebral system is more developed, and the size of the abdomen is proportionally diminished. In birds, the abdominal functions are less necessary still. In the *Herbivoræ* the abdominal secretions and excretions are very copious, because their internal organs are very large; while the *Carnivoræ* are as remarkable for the deficiency of both.

"Men, whose abdomen is either prominent or collapsed, approach very nearly the two extremes of herbivorous, and carnivorous animals. The first class, *abdominaux*, eat little at a time, but eat frequently; they digest almost continually, sleep much, and pass a sweet and tranquil life; but on the contrary, men, whose abdominal apparatus is small, compared with that of the head or chest, eat greedily, have imperfect digestion, form little chyle, and are dry and thin. It is not easy to conceive how physiologists can maintain, and even to the present hour, that a prominent abdomen, and great corpulency, are evidences of general weakness, for, say they, cellular tissue, and lymphatic fluids inundate all the organs, confine and impede their functions. But, besides that this explanation is altogether mechanical, it is not applicable to any organ, nor supported by any fact. It is easy to see, that the debility arises from the cerebral and thoracic systems, not from that of the abdomen, which is peculiarly active and predominant, which elaborates much chyle, and whose secretions are very copious. For how can one imagine, if we reflect for a moment, that large organs, which are in constant action, and whose products are considerable, have little energy. The abdominal viscera are subject to the general laws of organism; the more they are exercised, at the expense of others, the more they augment in relative size, and, by consequence, in relative energy; so that we can conclude, as we have done with respect to the thorax and head, that the relative size of the abdominal viscera indicates the relative energy of their functions." 103.

This, then, is the essence of Thomas's theory of temperament, and it may be submitted to the reader, whether it wears the attractions of a novelty. It is nothing more than the *doctrine of proportion*, which has been maintained from Hippocrates to Cabanis, and upon which, in fact, almost every division of this subject has hitherto rested. If any of the four constituents of the blood varied in quantity, this variation was made the origin of a new temperament. This opinion of Hippocrates, which was already as much a fancy as a fact, was stretched still further by his followers, and in place of limiting this variation to the elements of the blood it was extended to the general materials of the whole system; hence, we had the *muscular* temperament, if the muscular system prevailed, the *bilious* when the bile was in excess. But all this was *proportion* and nothing else, and when we are now told that we are getting a new system, and that the "author's astonishment was extreme" in finding so many accredited errors among writers on this department of physiology, we can neither say much for his modesty, nor participate in his surprise. It seems to us a distinction without much difference, whether we ground our doctrine of temperaments upon the predominancy of certain tissues and fluids, or of certain organs into the composition of which these tissues and fluids enter. The fundamental principle of both doctrines is *proportion*, and the arguments, which may be advanced in defence of the one, will equally apply to the maintenance of the other. If predominance of bile made the constitution bilious, or of blood sanguine, was not the disproportion, or preponderancy of either fluid the cause of such constitutional differences? And if the muscular, or nervous tissue prevailed, was not the excess of these tissues over the other textures of the system the reason why the constitutional habit was in the one case denominated muscular, and in the other nervous? If this be granted—and Dr. Thomas will not be likely to deny us a concession which it were absurd to refuse—we ask wherein differs an arrangement from either of the preceding, which takes systems of organs for the ground-work of its divisions, in place of systems of tissues or of fluids? If the brain preponderate over the lungs and the liver, the *cranial* temperament is formed; if the lungs over the liver and the brain, the *thoracic;* and if the liver over the brain and lungs, the *abdominal.* All this is a matter of mere preponderancy, of simple material proportion. The parts chosen for weight and measurement, it is true, are different, just as different as systems of organs differ from systems of tissues, or classes of fluids; but the principle of all these doctrines is one and indivisible, and the only merit which M. Thomas possesses, is that of having made a new selection of parts and organs for the application of an old principle.

In prosecuting this view of measuring energy of function by size of organ, the author enters upon *Phrenology*, and gives us a laborious *inch and line* description of the mental faculties, examines with great minuteness the exterior of the thorax in order to discover the volume of structure which it encloses, concludes his mensuration by giving us the admeasurement of the abdomen, and then sums up the whole with the following sentiments:—

"After having shewn how the cranial, thoracic, and abdominal organs, which nearly constitute the whole animal economy, form three very distinct groups, with reference to structure, situation, and function; having demonstrated, how in a state of health, the relative size of an organ is a fair index of its energy; and having, finally, examined the means of estimating, more especially in man, the development of the viscera, by an examination of the cavities within which they lie, we now pass on to consider in succession the effects of the predominance of the cranial, thoracic and abdominal organs, from which the different temperaments or constitutions arise." 130.

Before pursuing Dr. Thomas further, some critical observations upon this mechanical system of metaphysics will be indispensable. And first we would congratulate the profession upon this important *discovery!* The mysteries of function are now no longer to be talked of, the perplexities of metaphysics are now no longer to be encountered. The carpenter's rule and the school-boy's compasses have removed every difficulty! Mind may be measured by the foot, function by the yard, and the force of passions, the most unlimited, can be subjected to Gunter's scale! It was, certainly, once thought, that man was as much a *binary* compound as a *biped*, and that his material ingredient was not only inferior, but in all things subservient to his moral principles. But now it appears, that moral principle is the product of material structure, that the size of an organ is the measure of its function, and that for every square inch of solid matter, we have a certain forthcoming of vital power! Mensuration being then the basis of metaphysics, anatomy must be studied by the Casuist, before judgment can be given upon cases of conscience; and, by reading the culprit's scull, phrenology can ascertain without the hazard of perjury, or the subtleties of law, not only whether he be a *thief* or a *murderer*, but whether he have stolen ten, or ten thousand pounds. This is, indeed, no ordinary discovery;—verily, it is an improvement of vast magnitude even upon Gall and Spurzheim!

To be serious—is it actually the fact, that such as have great heads have great intellects, and that those, whose *enbonpoint* is considerable, have considerable appetites? Is it true that the largest liver secretes the most bile, or that the largest lungs arterialize the most blood and consume the most oxygen? To answer these questions, is to decide upon this theory. We admit, that in tracing animated beings up from the zoophite to man, the nervous system is in general on the increase as we ascend, and that the functional perfection of this system maintains some general ratio to the complexity of its mechanism. All this we are prepared to admit. But is the gradually increasing size of the *instrument* any proof of the gradually decreasing power of the *agent*; or is the mere circumstance of an organ's progressive development any argument against a corresponding progressive augmentation of its presiding and acting vitality. Let it be supposed, for the sake of illustration, that man is a compound being, that his materialism is under the superintendence of an intelligent principle, and that the functions of this materialism are the aggregate results of their co-operation, is it not reasonable to expect, is it not what would be anticipated, that the degree of organic life or presiding principle will bear some proportion to the quantity of organic matter or subordinate instrument, and that, since the presiding principle works only through the subordinate instrument, just as the instrument works only by the power of the agent, the agent's influence must generally limit the size of the instrument, and the size of the instrument must generally regulate the measure of the agent? Pure animal function never was, and never can be performed without the instrumentality of matter. It is only through such an intervening and co-operating medium that vital principle manifests itself in our present state of life, and it is neither fanciful nor fanatical to believe in the independent existence of mind, while at the same time it is maintained that, in a compound creature like man, both the energy and extent of his every function depend no less upon matter than upon mind. As the scale of organization ascends, the scale of life ascends along with it; texture becomes more minute, function more complicated, and the varying habitudes of different animals require corresponding modifications of structure. Muscular strength is required by one, acute sensibility is indispensable to another; the respiratory functions must predominate in this, the cerebral in that order of beings. Thus is it that the work to be done regulates the dimensions of the organ by whose instrumentality it is to be effected, and the size of the organ bears *in general (but by no means universally)* so close a proportion to its amount of vital principle, that, forgetting the agent altogether because unseen, this correspondence between size of instrument and perfection of function has induced many to ascribe all the phenomena of life to corporeal organization, because it alone is palpable to the senses. Life is thus degraded into a sophistical inference, and the æthereal chain by which man is linked to immortality is cut asunder!

The brain of the horse has been estimated at the five hundredth part of its body, while that of man stands as high as the thirty-fifth; but this fact goes no further than to shew, what no spiritualist ever denied, that the vital principle requires an increase of instrument when it is loaded with an increase of work. This is a reasonable requisition on the part of Nature, and a reasonable inference on our part; but to maintain that function is the uncompounded result of material structure, that thought is as much a manufacture as urine, and that passion can be placed between the compasses, or measured by the yard-stick are most unwarranted inferences, alike hostile to the interests of medicine and morality. The liver of the fœtus is its largest viscus, yet are the hepatic functions most active during fœtal life? The nervous system of the Ourang Outang is as large in proportion as that of man, yet are his moral principles as refined, or his mental powers as developed? Is it not well known that the *lean kine* of Pharoah ate more than the *sleek and well-favoured;* and is not the same fact as certain among ourselves? Has the Elephant, by having a much larger brain than man, any chance of outrunning him in the march of intellect? Is the muscular system of the common Maggot extraordinarily developed, yet is there an animal in being more active for its size? Are individual or national differences to be explained on such principles? Have the most gifted individuals the largest heads, or the greatest *blockheads* the smallest? Are the English more remarkable as a nation than the French for intellectual organs; or do the French exceed the English in that of self-esteem? Why are the Spaniards distinguished for treachery, or the Italians for love; and why are the passions of the Laplander nearly as cold as the snow he treads upon? How can external measurement of the chest ascertain whether its size depend upon hypertrophy of the heart, or of the lungs, or of both; and how can the exterior of the skull discover the peculiarities of cerebral structure, when no correspondence is preserved between its external and internal surfaces? These are merely a tithe of the interrogations we feel disposed to put to M. Thomas, and when these shall have received a satisfactory answer out of his system, our veneration for *Materialism* will be greater than it is. It is true that a *foot note of six lines* has been appended by the author, to a work of *two hundred and fifty pages*, with the view of rescuing his character from such a creed; but when we add that four of these lines contradict the other two, that the six together assert nothing, and that the motto of the volume is the following extract from Dupaty—"*That the moral man lies hid in the physical man*"—this *laboured* marginal commentary, we apprehend, will scarcely save the text from heterodoxy.

It is certain, then, that none of the theories which have been examined, not even that of M. Thomas, will account for every temperament. In the construction of the most of them an ingredient of vital importance has been virtually excluded, and until it be introduced, unfashionable as its introduction may be, we expect no purer light to emanate from such gross materials. Some of them have certainly revealed many facts and have removed a few difficulties, and to deny or resist their application to a certain length would be to fall into the error which it is our endeavour to chastise. To say that the solids may be relaxed, or that the fluids may be disproportioned; that the nervous system prevails in this, and the sanguineous in that constitution, or that one organ acts with more energy than another, is all very just, is what the natural philosopher might anticipate from a machine so delicately adjusted, and so easily deranged. But we maintain that all this is nothing higher than Mechanics, and until it can be shown that the human body is a mere machine, given up to the sole government of mechanical laws, such principles are no purer than unmixed materialism, and are totally disqualified to account either for the origin, or action of half the temperaments which exist. How often has one disastrous event metamorphosed the entire constitution of an individual, and reduced him who was superlatively sanguine into a melancholy recluse? How frequently are the passions, prejudices, and prepossessions which distinguished our early years, wholly altered or materially changed, when we arrive at a more advanced period of life? The enchanting and gilded thoughts of infancy vanish before the chaste and steadier light of manhood; and the sanguine anticipations of youth are obscured or buried in the clouds of age. A misty morning sinks us into melancholy, a transient sunbeam rekindles hope, and the dream of a night, or the success of a day so changes our condition as not unfrequently endangers our identity. Horace, speaking of the mutability of man, says—*nil fuit unquam sic impar sibi*—and Dryden's lines—

> A man so various, that he seemed to be
> Not one, but all mankind's epitome.
> Stiff in opinion, always in the wrong;
> Was every thing by starts, and nothing long;
> But in the course of one revolving moon
> Was chemist, fiddler, statesman, and buffoon.
> Then all for women, painting, rhyming, drinking,
> Besides ten thousand freaks that died in thinking.

are applicable to thousands besides the author of the *Rehearsal*.

In all these cases and such as these, both the structure and size of organs continue very much the same; there is something prior to mere corporeal organization, there is a vital principle, a spiritual agent to which the body is subservient, and which in our view is more efficiently operative in the formation of temperament than any, or every mechanical difference. It is true that the human body is a machine; but it is a machine endowed with life and fitted for living functions. It is true that the laws of chemistry and mechanics operate within it, *because* it is a machine; but then they operate in a peculiar way and under the direction of peculiar principles. *Life* and *mechanism* are, therefore, in close union in our present state of being, and every function which is carried on, whether it be that of thought, or loco-motion, is conducted by *a compound power, which is the result of life working upon suitable machinery*. The metaphysics of this subject have been sadly excluded from its consideration, and the science of life has been conducted with the level and the plumb-line. Phenomena, which are essentially metaphysical, have been traced to material causes; chemistry has been tortured for the solution of difficulties it was never adapted to remove; astronomy has been consulted in quest of light; and man has been studied, strange as it may appear, through the science of the stars! If the habit were irritable, it was because the fluids were irritating; if passive, it was because they were mild; if the temperament were moist, it was because the solids were deficient; if dry, it was because they predominated; if the functions were languidly performed, it was because the fluids were unstimulating and unfit for circulation; but if actively, the hydraulical department of the machine was in good working order. Such clumsy physiology might have been tolerated in the days of Paracelsus, but it is inexcusable in ours; and it ought to chastise the present boast of talent to have it said, that one of the first writers of our age among the French, and a distinguished Encyclopœdia-maker among ourselves, have advocated this crude philosophy without a syllable of improvement, and have sealed it with the arms of the nineteenth century.

In the inferior animals there are no such varieties as in man. One class or genus, no doubt, differs in its habitudes and dispositions from another, and there may occasionally be discovered some shades of difference among individuals of the same species, but these shades are very indistinct, and do not authorise any differential arrangement. Rousseau has, indeed, said that the monkey was only a savage man, and Linnæus has classified us with *Apes*, *Lemurs*, and, what appears even a stranger association still, with *Bats;* but as Materialists themselves do not now seem proud of such society, it is unnecessary to remonstrate with the taste of an arrangement so incongruous. *L'homme ne resemble qu'à l'homme luimême.* In our species alone are recognizable such distinct varieties, and the well-known fact, that these varieties so multiply as civilization and mental refinement increase, that the same nations and individuals differ as much from themselves at different periods as they do from others, is decisive that more importance should be attached, in the investigation of this subject, to the moral system of man, than it has been hitherto fashionable to ascribe to it. The young Savage of Aveyron had not been absent many months from the mountains, before he suffered three severe attacks of disease, and had changed very materially in taste and habit. How different was Rome in the reign of Numa from what she was during that of Augustus! At first, quiet in disposition and moderate in desire, she was satisfied with limited authority and innocent enjoyment; but her passions burned as her prospects brightened, feuds became numerous, factions formidable, a spirit of immoderate ambition began to wanton in the arms of luxury, and her whole moral character was ultimately changed. And what a contrast is presented in the eventful history of Wolsey, between the contented school-boy, while conning his master's task, and the disgraced Cardinal while mourning over the disastrous career of his immoderate ambition! The problem of Condillac—"the organization of the physical man being given, his moral constitution is required"—can never receive from these principles a satisfactory solution, and the sentiment it conveys is neither safe morality nor sound metaphysics. The intimacy which subsists between mind and matter in the human system is mysteriously close, but mind can exist without matter, as well as matter without mind. Their union is conventional, not necessary. The qualities of the one are as various as those of the other and at the same time as distinct, and while there is no evidence to shew that an inherent tendency to disease or annihilation is one of the mental properties, there is, as has been already argued, convincing proof that its original constitution in different individuals may be very different, and that this difference may, from its connexion with and influence over organic function, impart a constitutional peculiarity to systems in other respects the same. It were as difficult to maintain that the minds of *Shakespeare* and of *Chrichton* differed in nothing from those of *Louis the 8th* or *Charles the 2d*, as it were to shew that *Dominie Sampson* and *Dumbie Dykes* were twin brothers. Perhaps two individuals in all respects intellectually similar never have existed, and since mind is a creature as much as matter, although different in essence and obedient to different laws, why should it not be equally subject to original differences? The talented youth may degenerate from misfortune or misconduct, and the most unpromising powers may be improved by exercise. Climate may make the courageous timid, and the mild may become ferocious through adversity. Want may palsy the hand of benevolence, and a favourable crisis may develop springs of action and lineaments of character which might have otherwise remained unnoticed, because unknown. All this may happen, but this is not sufficient to account for the ten thousand forms of metaphysical being, and the ten times ten thousand shades of talent and of taste; and so long as we know that man is a compound, that his component parts are different, that he exhibits during every period of life properties pertaining to each department of his system, that these properties differ in different individuals, and that their difference can only be accounted for on philosophical principles such as ours, we ask, and we put the question home to every friend of materialism, why perplex the subject by confining ourselves to one department when both should be examined; why, by striving to explain metaphysical phenomena on physical principles, leave most interesting questions without answers, and why study a compound being, and a being whose every action is a compound action, by laws which can only apply to one hemisphere of his constitution? We might, with as much prudence of system and as much prospect of success, learn geography upon *half* a globe! Who could ever master the localities of America by travelling through the Continent? What Logician would hazard a conclusion in the absence of his *major* premise; and what Historian, who was not somewhat more than courageous, would write upon the constitution of a *mixed* government, while wholly ignorant of the prerogatives of the *King*? If man will not be studied with reference to his constitution he can never be understood, and although a few of the problems of life may be worked with the aid of chemistry, dynamics, hydraulics, and of algebra, many must be abandoned as inexplicable mysteries, or receive their solution from the labours of Locke, Reid, and Stewart.

There are, then, *two* sets of causes of temperament—*corporeal* and *metaphysical*, which, while they are widely different in their nature, by their action and re-action on each other produce very similar effects. The preponderance of a certain organ, the disproportion of a certain system, the susceptibility of one tissue above another, the peculiarities of climate and of education, the habits of society, and the varying changes of life, pertain to the first class and are highly influential; but there are other agents no less powerful, which are purely metaphysical, and which result from the different states and proportions of the mental powers. The imagination may be constitituonally weak, or active; the perception may be acute, or dull; the judgment may be impotent, or strong; the passions may be cool, or ardent; the imaginative faculty may prevail in one mind, the reasoning faculty in another; love of power may give a cast to one individual; thirst after fame may mark the character of the fourth: and as certain corporeal actions are directed by certain moral causes, not only the functions but the form of man must be considerably under their control.

It is not intended to be argued that the conditions of the body will always correspond with those of the mind; they certainly will not. It is only maintained that the operation of certain moral principles will favour the development of certain physical powers, just as the preponderancy of certain physical powers will favour the development of certain moral feelings; and it is this action and reaction, this interchange of influences which we wish to insist upon, and which in our view can only be studied or understood under the guidance of our principles. We are, therefore, equally opposed to Niederhuber, who ascribes all to difference of vital power, and to Thomas, who attributes all to difference of organic structure; neither system being singly sufficient. Both systems operate in the discharge of general functions, both systems influence the functions of each other, and, consequently, both systems should enter into our views of temperament. Metaphysics in medicine is like physic in *a farm-house*, it is seldom thought of. If the body be depressed it is excited, if weak strengthened, if strong weakened, if cool heated, and if hot cooled. The veriest machine that was ever wrought could not be treated more mechanically. The blacksmith tempers down his iron if it be too hard, and reduces his spring if it be too strong; the engineer opens his safety-valve if his steam be in excess, and throws on fuel if greater power be required. With the blacksmith and engineer this reasoning is correct, for the mechanism, with the actions of which they have to do, being palpable to the senses and obedient to the laws of mere matter, can be fully judged of by the senses and subjected to these laws; but with the physician it is seriously different. He is concerned with a mechanism which is at the disposal of a presiding principle, which gives to it varieties of function proportionate to the varying constitution of its own nature; while the agent in turn is itself subject to the reaction of the instrument through which it operates. This important, this fundamental fact should never be lost sight of.

Mental constitution must always be studied in connexion with corporeal mechanism. A true knowledge of the one is incompatible with total ignorance of the other. Man is but half explored when the anatomist has finished his dissection. When his knife has revealed every nerve, when his microscope has disclosed to sight the hidden mysteries of structure, and when his curiosity has exhausted its resources by ultimately reaching the rudimental molecules of organization;—we ask what has he done—what has he discovered? Has he found out any thing like life? Has he revealed the seat of thought, or the granary of memory, or the throne of judgment, or the gallery of imagination? Has he drawn the veil from off the fountain of action, or has he traced up function to its source? In the origin and insertion of his muscles can he detect the cause of their contractility; or will chasing a nerve to its most capillary twig shed a ray of light upon the mysteries of its office? To know that the liver is composed of glandular acini, that the brain is a congeries of agglutinated globules, that involuntary differ from voluntary muscles in being made up of fibres which are not parallel but anastomose, does not add one idea to our scanty store of information upon the elaboration of bile, or the phenomena of mind, or the origin of motion. After collecting every circumstance and comparing every fact, which the accumulating labors of centuries have disclosed on glandular anatomy, are we one jot the wiser on the subject of secretion, and can we assign a single reason, having any degree of plausibility, why the kidneys should not secrete bile, or why they do secrete urine; and how one common fluid, which is apparently similar in whatever organ met with, can furnish the richest variety of substances, and can deposit these substances with such unerring precision, as to time and place and quantity, that muscle is never secreted where bone ought to be laid down, and skin is never formed where either would be necessary? Unassisted mechanism is a deaf oracle upon all these subjects. How often has she been, how often is she consulted; but she answereth not, and never will, never can answer? The worshippers of materialism may continue to call upon her, and hecatombs of subjects may be yearly offered up to her, and the incense of phrenology may ascend to her from every surgery upon the Continent, and our youth may be made to worship her from Dan unto Bersheba, and all such idols of fanatacism as independent life, immortal principle, aura divina, spiritus æthereus, may be immolated before her upon the shrine of liberal sentiment as a vindication of her supreme divinity, and the God of the Creation may be sent to herd, like the Babylonish King, with Lemurs and Bats as a proof of the superior enlightenment of the present age; but, as for us we are still too strongly wedded to the venerable worship of our forefathers, or, if the phrase be more palatable, too strongly warped within the prejudices and prepossessions of their antiquated dogmata, to see any thing in a system, constructed out of such unworthy materials and cemented by such untempered mortar, which should induce us to abandon views authorized by revelation, supported by reason, established by metaphysics, uncontradicted by any facts and confirmed by every day's observation. *Nescio quomodo inhæret, in mentibus quasi sæculorum quodam augurium futurorum; idque in maximis ingeniis, altissimisque animis et existit maximè, et apparet facillime.* Such is the testimony of Cicero; and we do esteem that bastard independence which can look upon non-existence with apathetic coolness, yea even that superlative degree of it which can convert a theme so solemn and so sacred into a subject of profane ridicule and of reckless reviling, as the last and lowest evidence of a debauched and degraded mind.

"From what we have seen in the first part of this work, temperaments or constitutions are the varieties which result from the different proportions of the three great cavities. Now we can distinguish in man seven temperaments. The first is the *mixed*, or that in which the three cavities are proportionable; the second, third, and fourth are the *cranial, thoracic*, and *abdominal* temperaments, as the cranium, thorax, or abdomen preponderates, and the fifth, sixth, and seventh are binary compounds, as the cranium and thorax preponderate over the abdomen, or the cranium and abdomen over the thorax, or the last two over the first. These are denominated the cranio-thoracic, cranio-abdominal, and thoracico-abdominal temperaments." 132.

It is evident that as the accuracy of this division must be proportioned to the truth of its principles, little dependence can be placed in it. The *mixed* temperament is regarded as the best, and the Apollo of Belvidere is given as its most perfect representative. In such a constitution there is nothing in excess. The brain cannot be the seat of passions too violent, although it may exhibit them all; the intellectual faculties are sufficiently developed, and do not allow themselves to be misled into the labyrinths of hypothesis, or the uncertainties of conjecture; the blood is neither too "fibrous nor animalized," the abdominal functions are freely discharged, every member can exercise with the greatest ease every necessary movement, and the physiognomy represents in every feature that perfect proportion for which the entire system is distinguished. "The mixed temperament is very general among the French, and it is most frequently observed between the ages of 20 and 45, while every organ is in a state of maturity."

We wish not to contend the right by which M. Thomas ascribes to his countrymen the *beau ideal* of bodily and mental perfection. If such partiality be not well founded, it is at least natural. But we must maintain, that as far as history and our own experience go, moral worth and corporeal beauty are by no means so closely united. Homer describes Helen as violently passionate; Cleopatra was enthusiastically fond of Anthony; and the unfortunate Queen of Scots was equally distinguished for her imprudence and her charms. Minerva was never painted by the ancients as Venus, nor Mercury as Bacchus; and the Grecian youth, who fell in love with his own person, will never be cited as a model of intellect. It is unnecessary to describe the remaining temperaments minutely. Cataline, Tiberius, Brutus, Cassius, and almost every hero of antiquity; Pope, Pascal, Voltaire, Rousseau, and almost every genius of later times, are adduced as examples of the second, or *cranial* temperament. The statues of Pericles were said to be crowned with a helmet to conceal the disproportioned size of his head, and Teleclides, in his description of him, supports our author's theory, if a poet's portrait can be deemed a likeness;—

> "Perplex'd by business, by its weight depress'd;
> Now his huge head hangs silent on his breast,
> While from that head, in which ten men might dine,
> Loud thunders burst, of dreadful storms the sign."

This information respecting Pericles is to be found in Plutarch; but M. Thomas has not dealt so liberally with us, for we are left to discover where he has obtained the cranial dimensions. Hercules is the model of the *thoracic* temperament; which is common among the robust inhabitants of the country, and men of active habits. It is more favourable to physical than mental exertion. The *abdominal* constitution is more frequent in town than country, and, as might have been expected from our author, is described as more characteristic of the English, Dutch, and Germans, than the French. The *cranio-thoracic* is the temperament of usurpers, great conquerors, and, a combination which may render one class of the profession a little vain, of *good operators*. The *cranio-abdominal* temperament is said to belong especially to females and to infancy. But if "*mulier propter uterum condita est*" be a sentiment of any truth, the ventral temperament should be more appropriate to the former. Individuals of such a constitution are more attached to sensual pleasures than mental enjoyment. Lastly, the *thoracico-abdominal* is the constitution of the inhabitants of Asia and Africa, and is considered the most unfavorable form of temperament. When very perfectly developed, madness, idiotcy and imbecility are the ordinary consequences.

As our main object in this paper is to ascertain the nature and influence of temperaments generally, it is not necessary for this purpose to insist upon any division, much less to examine the merits of all those which have been proposed. The principal classifications of the ancients have been already noticed; those of Haller, Cabanis, Richerand, and Hallé have been mentioned, and that of M. Thomas is now before us. Our views admit of an arrangement different from any yet made public; but as a review is not the most suitable place for its introduction, we shall neither propose any new, nor yet implicitly follow any old division. Perhaps, for practical purposes, that adopted by Bostock is the best. "If to the four temperaments of Hippocrates we add, after the example of Gregory, a fifth, the nervous temperament, and bestow new appellations upon the other four we shall have the leading varieties of the constitution under the denominations of the *nervous*, the *sanguine*, the *tonic*, the *relaxed*, and the *muscular* temperaments." Of the above the *melancholic* and the *sanguine* are not only the most frequent, but seem to us the only original temperaments. The *nervous* habit, although the creature of society, and, therefore, artificial, is, however, deserving of distinct notice; for until our present habits of luxury and splendour give way to a simpler mode of life, it is quite certain that it will continue one of the most important, because one of the most common temperaments. Of the rich and fashionable, the luxurious and indolent, it is the almost constant companion. As to the points of difference between these three and the *phlegmatic* and *choleric* constitutions we shall speak hereafter. The sanguine is the temperament of the young, and most northern nations; the melancholic of the old, and of the inhabitants of moist climates. But since every system is distinguished for some peculiarity, these elementary simples are seldom found in an uncombined state. Hippocrates maintained that our temperaments changed as often as the season; that in Summer they were sanguine, in Autumn melancholic, in Winter phlegmatic, and in Spring partly sanguine and phlegmatic, making atmospheric temperature and corporeal temperament to correspond. But, without asserting so much, it may be said that scarcely are two bodies alike formed, or two minds alike constituted, and that many circumstances may occur during life to render totally dissimilar, what at its commencement had been virtually the same. The ardency of youth may be frozen by adversity, the despondency of age may be antidated by misconduct. The choleric may become phlegmatic, the melancholic sanguine. With all these states of system it is natural to expect different degrees of health.

In the *melancholic* temperament, which was called by the ancients *siccum*, because the solids were dry, the heart is moderate in its action, the pulse slow, and the functions of life sluggishly performed. The mind is tardy in resolve, but steady in action. Perception is slow, judgment sound, memory uncertain, imagination gloomy. Every event is examined in its dreariest view, trifling dangers are magnified by fear, hopeful prospects are obscured by despondency. The stomach and liver are easily deranged, the intestines are torpid, and it has been fashionable to regard the biliary secretion as diseased. The solids are supposed to be firm and constricted, the fluids scanty and viscid, and diseases of obstruction as dropsy, hæmorrhoids, jaundice, and affections of the brain as apoplexy, phrenitis, mania, are liable to occur. The melancholic temperament is most frequent in advanced life, and among people of sedentary habits. It seldom appears in youth, misfortune is its most ordinary cause, and being found frequently accompanied with visceral disorder, many have regarded it more as a natural κρασις, than as a symptom of disease. Works of the most laboured industry have been the fruits of this temperament, and minds of the highest rank have borne the burden of its influence. Rousseau and Zimmerman, Gilbert and Pascal, are illustrious examples of such as combine the most desponding views of life with the most luxurious means of rendering life happy; and in the delightful Tasso, and interesting Kirke White we have melancholy proof that the liveliest imagination, supported by the buoyancy of youth, can neither always avoid, nor counteract it.

In the *sanguine* habit circumstances are very different. The action of the heart is strong and quick, the pulse frequent and full, the functions conducted with energy and effect. The mind is ardent and aspiring, impressions are easily effaced and made, promises are more willingly entered into than performed, the taste is capricious, the fancy wayward. The judgment is not as sound as the imagination is active, the memory is more ready than retentive, perception is quick, love ardent but easily cooled, anger excited by trifles and by trifles laid. Sensibility is acute, irritability is great, fortune is enjoyed in her sweetest smiles, and adversity is suffered in her cruellest pangs. The solids are lax and the fluids copious. Susceptibility to disease is very considerable, and the diseases most readily induced are those of excitement. Inflammations of every degree, fevers of every type, hæmorrhagies of every organ. This form of constitution is general in youth and prevalent in manhood; but it rarely sweetens the vale of life, for experience is its deadliest enemy. Our knowledge of the past, while it makes the present less faithless, renders the future less promising. As the drama of human life hastens to a close, the scenery becomes less and less fascinating, things appear more as nature made them, the flame of hope sinks in the socket of disappointment, and ere the curtain falls the spell is broken. The sanguine temperament has been called by Prichard *constitutio Germanica* from its prevalency among the Germans, in the French and Irish it is very common, and some have maintained that it is less frequent in females than in males.

As the melancholic is the temperament of age, the sanguine is that of youth; but between these two constitutional extremes there are many points of difference, and many varieties of temperament have been formed to account for them. We have the *choleric* and the *phlegmatic*, the *acrid* and the *bilious*, the *moist* and the *nervous*. It must, however, be admitted that any intermediate varieties which exist are rather compounds than simples. The chief distinction between the sanguine and the dry is that the muscular system is more developed in the former; and the moist differs from the cold in having a greater stock of fluids. If the solids be denser and the nervous system more irritable than in the sanguine, then we have the choleric; and if we find less irritability and greater strength the phlegmatic habit is produced. We deem it, therefore, unnecessary to go into any description of what are mere combinations. The simples being once well known such compounds as may result from them it will not be difficult to understand, and although the phlegmatic and the choleric have been hitherto regarded primitive, they appear to us to be secondary formations. The phlegmatic agrees with the sanguine in having abundance of fluids, and with the melancholic in having little irritability; it is, therefore, the product of a certain mixture of properties pertaining to these two elementary habits; while the choleric to the irritability of the sanguine adds the pertinacity of the melancholic temperament, thus shewing that in the sanguine and melancholic exist all those general properties out of which subordinate peculiarities originate. The sanguine would be choleric if he were less fickle, the choleric would be sanguine if he were less pertinacious; the sanguine would be phlegmatic were he more passive, the phlegmatic would be sanguine were he more irritable. So far, then, as the formation of these four forms of temperament is concerned, two series of principles variously proportioned are sufficient; and as these proportions can be varied *ad infinitum*, the variety of secondary habits may be infinite.

As to the *nervous* temperament, it is merely the product of excess and luxury. Such a constitutional character is seldom found in rural and simple life. As the appetites are gratified desire increases, demands are made upon the expenditure of the system which it is impossible to grant without sustaining injury, and the nervous system being the most sensible to disorder is the first to betray manifestations of disease. While we oppose, therefore, the opinion of Darwin, who maintains that "by temperament (in general) should be meant a permanent predisposition to certain classes of disease," it is evident that, in as far as the nervous habit is concerned, its definition may be couched in still stronger terms, and it may be said that by the nervous temperament should be meant the first stage of constitutional decline. Certain it is that this habit is becoming every day more common. The list of diseases which Dr. Trotter enumerates as pertaining to it is truly formidable, and were some of the hints attended to which that very able writer has made upon the effects of dissipated life, nervous head-aches and hysterics would be much less fashionable and much less frequent. At present it is deemed vulgar to look healthy; the pale and sickly are alone interesting; and if our youth wish to be considered handsome or genteel, the bloom of health must be got rid of, head-ache must be frequent, and the turning of a leaf, or the tread of an insect must never fail to be followed by an evening's set of fits, or a day's indisposition! However profitable this fashion may be to one class of the community, and however enviable it may be held to have our lady's health hourly enquired after by fifteen or twenty visitors, it is devoutly to be wished that the present rage after fine figures and genteel faces, after morning head-aches and evening vapours, may not increase much further; otherwise every family will require its resident physician, or stand in hourly danger of being thrown into confusion through violent attacks and sudden illnesses. The wealthy and the noble, it is true, being alone competent to struggle with such a tax upon their constitution, are tolerably sure, as long as it is paid, that neither the pauper nor the tradesman can in this instance annoy them by treading on their heels; but if sickness be necessary to the acquirement of distinction, we may hazard the charge of vulgarity by thinking so, but we do think that the distinction is too dearly purchased. There is at least one consideration which should be of some weight with those who advocate present fashionable life. It has been asserted by a very competent judge, that *fortes creantur fortibus et bonis*, and although the privilege of making our constitutions as degenerate and contemptible as we choose may be insisted on, there is neither law, right, nor reason for inflicting upon posterity miseries, the danger and extent of which are to us equally unknown.

Family likenesses are frequent and familiar, slight hereditary deformities are not uncommon. While Dr. Gregory was visiting in a distant part of Scotland, he met several people remarkable for a peculiar form of the nose, resembling that of the Grand Chancellor of Scotland in the reign of Charles the First; and upon making enquiry they were found to be the descendants of that nobleman. The family of the Le Comptes were hereditarily subject to blindness. Before the age of sixteen their sight was tolerable, but after that period it gradually declined. Now, if temperament be something like a tendency to disease, and if disease be hereditary, unless a parent have been denied by nature a parent's feelings, he surely cannot be told with indifference, that while he is by rioting and wantonness destroying his own system, he is also infusing into the stamina of his future progeny debility and disease. Some families are victims of the same maladies from generation to generation. One is preyed on by consumption and another by scrofula; one is subject to madness and another to epilepsy; gout is the scourge of this, hydrocephalus of that family. Frequently, no doubt, the circulation of such currency is beyond our control. The stream may be contaminated ere it reach us, or the hereditary affection may be such as defies exertion. In such cases our only duty is submission, for nothing is expected where nothing can be done; but when we have at our own disposal whether our children shall be useful or a burthen to society, whether they shall be a curse or a comfort to themselves, reason, religion, affection, interest and duty are a sample of the motives which should influence our choice.

In the *sanguine* diseases of strength and activity are prevalent; in the *melancholy* visceral and vascular obstructions; and in the *nervous* affections of excitement and debility. These propensities depend upon two sets of causes. The metaphysical composition of the *melancholic* is more remarkable for steadiness than activity, for perseverance in pursuing an object than for love of enterprize. So is it with his corporeal mechanism. His nerve is less sensible than firm, and his fibre more disposed to continue than commence disease. When an exciting cause is applied to such a system, it may either be entirely resisted, or only partially succeed, and an attack, which would have committed destructive ravages in a more susceptible habit, will by it be comparatively disregarded. When the excitement, however, has had effect, and morbid action has commenced, the same principle by which it was at first resisted now favours its continuance. Slight derangements are less common than extensive disorganizations. Symptoms are slow in progress and moderate in degree, the general system is little affected by local disease, and visceral action of most destructive type may be working its silent death of parts the most important to life, and in consequence of the few sympathies by which it is surrounded little may be suffered, and still less feared. Hence are daily met with in the bodies of such individuals enlargements, hardening, and abscesses of the liver—hypertrophy of the spleen—adhesions of the intestines—thickening of the mucous membrane of the stomach—obstructions of the mesenteric glands—consolidation of the lungs—ossification of the valves of the heart and coats of the blood-vessels. In all these and other such affections length of action compensates for energy, chronicity for acuteness, and extent of ultimate mischief for the reluctance to activity which at first was manifested.

In the *sanguine* all is nearly opposite. The mind is sensible and active, impressions are easily made and dissipated, pain is exquisitely felt, and pleasure enthusiastically enjoyed. In this constitution susceptibility is the leading character both of mind and body. The sensibility of the body renders prostitution of the mind more easy through temptation, and the sensibility of the mind renders disease of the body more easy through excitement. The strongest tendency to physical and moral evil is thus combined in the same system, and although there are counteracting causes which may weaken the tendency of the former, that of the latter is too frequently successful, having weaker guards against greater dangers. Temptations trifling in the abstract are here formidable, and causes of disease, which in the preceding temperament would have been successfully repelled, do here more certainly take effect. The slightest mental agitation inflames the blood and shakes the system, and the slightest aberration of the body from a state of health irritates the mind. As the diseases of the melancholy are chronic, those of the sanguine are acute. Inflammation once begun rapidly proceeds, fever once kindled burns with an exterminating flame, and hæmorrhage once established requires but a few minutes to destroy life. Where so much susceptibility to disease is combined with such activity, the hazard is extreme if symptoms be neglected; but nature seldom exposes to danger so imminent without furnishing means for either its prevention, or removal. The same sensibility, which assists the exciting cause to the establishment of disease and hurries that disease forward to a crisis, gives a prominency to its symptoms by which they cannot fail to be observed, and thus affords early evidence as well of its extent as of its existence. Action being acute concealment is impossible; the features being fully pronounced, the face cannot be mistaken; and although the date of danger is nearly that of the attack, the warning is given when the first blow is struck.

In the *nervous* temperament tendency to disorder increases with a corresponding decrease in the powers of resistance. Mind and body, as it were, are more intimately intermixed than usual, and action and re-action are, therefore, greater and more frequent. The natural susceptibility of the sanguine is in this habit morbidly accumulated, and the tone and strength of the melancholic are almost altogether lost. The imagination is wild, the judgment easily deranged, the passions strong and easily excited. There is nothing so extravagant which may not be fancied, nor so improbable which may not be believed. The Impostor Mesmer was well initiated into the mechanism of this temperament, and nervous people are very generally selected by such jugglers, as the safest whereon to practice their deceit. The fibre is lax and unresisting, the nerve is unstrung, the heart is irritable, the circulation weak but rapid. Dr. Trotter discovers in deranged sensations and inverted sympathies of the great sympathetic the source of all nervous disorders. Whether the causes be moral or physical, they are supposed to exert their influence on this portion of the nervous system,—

"Whose office directs the most important operations in the animal economy, and binds together one great circle of feeling, actions and motions both distant and opposite. Hence a concourse of symptoms of the most extraordinary kind, that invert the usual functions of so many viscera, suspend their power, or give to them new movements, by which means a train of false perceptions occupies the mind, and ideas the most monstrous and incongruous supplant for a while all rational thought. In this reciprocal action between body and mind, in whatever part of the system disease commences it is quickly communicated to all the others.—Thus, in a dyspeptic condition of stomach, such as attends nervous complaints, it is not the muscular fibre alone of that organ which is to be considered as diseased, but every gland and pore, exhalent and follicle which separates either gastric juice or mucus, and, consequently, all the fluids are poured forth in a vitiated state. The appetite will then be irregular, sometimes suppressed, sometimes voracious; the acidity will increase so as to become painful, the food will remain indigested, and uneasiness and inflation of stomach will succeed. Other viscera will by consent of nerves be also deranged in their respective offices. The pancreas, its juice and duct are affected. The liver will excrete the bile in quantity and quality both different from its healthy state, and the ducts will be irregular in conveying it forward. The peristaltic motion of the intestines will be inverted and inconstant, and constipation or diarrhœa be the consequence. Even the kidneys, more remotely connected, will discover indisposition by the urine being voided turbid or pale, in small or profuse quantity, sometimes with pain in the loins, ureters, bladder, testes, or mammæ."

The duration of nervous diseases is as different as their character. They may appear in paroxysms of a few hours length, or continue uninterruptedly for years; and wherever the nervous temperament exists in perfection, there will not only be a strong *penchant* to derangement, but considerable difficulty in removing such as has already formed. An opinion, which to us seems rather novel, is maintained by M. Thomas on the ratio between the physical strength of a part and its susceptibility.

"The stronger and more predominant an organ is," says he, "the more sensible it is to natural excitements; the more is it inclined to exercise. And as it is not easy for an organ, strongly disposed to action, to avoid excess in obeying its own inclination, it often becomes diseased. Thus, men, in whom the brain predominates, frequently fall victims to the excessive exercise of their passions and faculties, and become affected with melancholy and *les folies variees;* the thoracic are obnoxious to carditis, pneumonia, and acute rheumatism; and the abdominal to gastric and hepatic disorders. A weak organ, although little prone, on the contrary, to action, although little sensible to excitants, is not less frequently obliged to use immoderate exercise, because a thousand varied circumstances often accumulate upon it natural stimuli. It is thus that when the thoracic viscera are small their action is easily increased, and they become exposed to thoracic congestion and various forms of phthisis. On the same principle idiots and they, whose brains have little energy, become epileptic and apoplectic by a fright, a dream, or an immoderate exercise of these organs, faculties, and passions." 214.

It seems, then, that it matters little whether an organ be strong or weak, its susceptibility remains the same. If strong, it will be weak through excess; if weak, it will be overpowered by exercise. So much time has been already spent in criticising this *mensuration-system* that a very little more can be devoted to it; but we cannot avoid observing that our author is here treading upon dangerous ground. If there be any one truth in pathology more evident than another, it is this—that susceptibility increases as tonicity diminishes, and it is a fundamental dogma in dynamics, that the power of resistance is weakened by weakening the power of the resisting body. Now, if a temperament be the product of the excess of one organ or set of organs above another, of course the predominating organ or organs, which constitute this temperament, should form the strongest of the whole fabric, and should, therefore, be the last to allow the inroads of disease. If a muscle be much worked it becomes large and strong, but if neglected small and weak; if the lungs be moderately but constantly exerted they increase in size and force, and so, we believe, is it with every other organ and texture of the body. But to bear out M. Thomas's theory, the strongest must be the most, and the weakest organ the least susceptible, and not only so, but the strength of an organ's tendency to disease should be in an inverse ratio to its physical strength. To preserve consistency, then, first principles must be overturned, and the rules of science must be sacrificed to the whims of theory. If an organ be very susceptible to disease because it is weak, it is quite impossible that it also can be very susceptible to disease because it is strong; this is arguing in a circle, without cause or consequence.

When points are thus minutely examined their fallacy is soon discovered and easily exposed; but if a superficial view be taken of many parts in the *Physiologie des temperamens* they will appear plausible, if not true. Thus after endeavouring to prove in the first part that temperaments depend on organic and functional proportions, he endeavours to establish in the latter part that diseases are the consequences of these deranged proportions. When the habit is thoracic the diseases are acute, because the lungs are spacious and form a great quantity of blood, and because the heart is robust and circulates it actively; whereas when the temperament is cranial the brain becomes deranged from excess of action, but, as it is more obnoxious to inquietudes and causes of disturbance, its diseases may be as active, but they will be less regular. The great majority of affections, according to our author's doctrines, are acute. As they all originate in excess of action, they all depend on visceral congestion. The mode of cure which he recommends is, therefore, to diminish the exciting causes, and to moderate the action of the excited organs. To effect this in persons of the *cranial* habit the fatigues of study must be given up, passions must be moderated, exercise must be taken, extremes of temperature must be avoided, and a vegetable, or a very light animal diet should be used. When the congestion is great general and local bleeding are necessary, after which antispasmodics may quiet the disturbance of the nervous system. The diseases of the *thoracic* habit being acute, the mode of treatment best adapted to them does not require specification. The *abdominal* temperament, he says, may be avoided or diminished by frugality, by indulging the passions, by studying closely, and employing active exercise. This regimen may succeed while the system is young, but if neglected to advanced life circumstances as numerous as they are irresistible will counteract its tendency, leaving nothing to be done but to prevent its increase and guard against its effects.

All this appears simple and probable enough *à priori*, and the facilities which it holds out to the changing and modifying of temperaments are encouraging. To convert the abdominal into the cranial temperament all the functions of the former must be discountenanced, those of the latter must be exercised. The mind must be stimulated by study, the passions must be strengthened by gratification, and such food must be taken as will inflame the blood already formed, rather than add to its quantity. Again, to convert the cranial into the abdominal temperament the reverse system must be pursued. The center of action may be thus variously changed, sensuality may take the place of intellect, and every bloated Bacchus may be made, according to our taste, Mercury, Orpheus, or Apollo. This is at all events a convenient system. The man, who is urged by misfortune into poverty, may take advantage of his circumstances, cultivate his cerebral powers, and convert necessity into a school-mistress. Every pauper, when he can do nothing else, may thus be made a Newton; and every workhouse may be converted into a parish-college!

Galen has said that the body of man is like Vulcan's forge, to which the anvil and hammer and bellows and fire were all equally necessary. So in man, if one system be removed or one organ engross the activity of the whole, there can neither be harmony nor health. Such gross disproportion Nature has never been guilty of. She may make one organ somewhat stronger than another, or one tissue less susceptible than another. But that, which was constitutionally the strongest organ, may be rendered soon after birth the weakest, by not being introduced into circumstances favourable to its action; and the natural plus-susceptibility of a tissue may be lowered during life into moderation or deficiency. Original constitution may thus be altered by accidental causes, the center of action may vary its position from the head to the heart, or from the heart to the kidneys, and differences may occur after birth, in the powers and proportions of the system, infinitely greater than any which existed previously.

As the diseases, to which the *melancholic* are prone, are more chronic than acute, and as the individuals, in whom this temperament prevails, are not remarkable for irritability, whatever treatment we pursue should be regulated under the knowledge of these circumstances. In many of them the most effectual means for their removal are moral and metaphysical. In hypochondriacism and mania, two of the most obstinate diseases to which man is subject, but little aid can be expected from medicine alone. Much depletion is not well borne; much excitement is improper. The body must be acted on through the medium of the mind, every amiable affection must be exercised, every social tendency must be stretched, amusement must be presented under every form, and every thing must be guarded against which might tend to encourage the favourite hallucination by which the mind is led astray. When medicine is given, it should be given cautiously; purgatives and local bleeding are the safest evacuants, narcotics are, perhaps, seldom, and ardent stimuli never useful.

In the other diseases of this temperament, the indications of cure must be formed and followed according to individual circumstances: but, as a general remark it may be observed, that the obscurity and apparent mildness of the symptoms should seldom be considered indicative of either weakness of action, or deficiency of strength. Such symptoms are the effects of such a temperament, and the knowledge of this fact may prepare us for consequences which we might not otherwise expect. The symptoms are mild because the sensibility is low, their progress is tardy because the action is chronic, and the voice of danger comes too late because the vital functions being gently assailed are only gradually disturbed. This insensibility as to disease is usually attended with considerable passiveness as to treatment. The operation of medicine is often weak, the effect of treatment slow, and the physician is obliged to encounter the same resistance which the disease had at first to contend with, but has ultimately overcome.

These remarks reversed will apply with tolerable fidelity to the treatment of disease in the *sanguine*. Ardent in spirit and irritable in structure, weak impressions produce strong effects, action is easily kindled and rapidly proceeds, organization is soon destroyed and disease requires but little time to reach maturity. Treatment in such cases must be adopted early, and vigilantly pursued. Depletion must be liberally employed when necessary, mental excitement and physical stimuli religiously avoided when injurious. Every function is easily impressed, medicines operate in small doses, and the patient is soon saved from danger, or soon hurried to his grave. As the symptoms in the melancholic were chronic, they are here acute. If pain be inconsiderable, action cannot be high; if symptoms be moderate, the danger cannot be great. In affections of this temperament, therefore, there is more to be feared from a sudden attack than a lengthened action, there is less deceit than danger, less to be dreaded from officiousness than neglect.

"It has been (says Trotter) unfortunate for the medical profession, as well as patients themselves, that persons labouring under nervous disorders have too much expected from the prescriptions of the physician and shop of the apothecary, what is only to be obtained from their own caution and circumspection. We thus find most of them ready and greedy to swallow every medicine that is recommended, but stubborn and intractable in all that relates to breaking in upon the established habits and customs, whether of luxurious living, depraved appetites, indolence of body or mind, or vicious indulgence of any kind inconsistent with health. Many of these habits, it is true, are so far interwoven with the constitution, as to make some changes almost impracticable; but as indisposition is so frequently brought on or aggravated by the improper conduct of the patients themselves, the physician cannot be too much on his guard in demonstrating to them all what belongs to their own government and demeanor. The medical adviser, therefore, who observes the most disinterestedness towards his friend, will often be the first man to be dismissed; while the selfish dissembler, however ignorant, will become a favourite, and engross the emolument. On such an occasion the virtuous mind of a liberal physician will know where to look for approbation."

To prevent diseases is the best way we know to cure them. When habits of idleness and luxury are firmly formed; when the system has become inured to favourite stimuli; when the drunkard cannot do without his bottle nor the glutton without his joint—the prospect of reformation is forbidding. Associations ardently cherished are to be broken up, and passions, which have ever proved the strongest in our nature, are to be overcome. To drink water instead of wine, to be limited to a simple dish, to labour several hours every day, to abandon every haunt of indulgence, to retire soon and rise early, are changes of the most repulsive character, and are more easily approved of than effected by the irresolute voluptuary. The *nervous* temperament is not natural to man as is the sanguine; it is the creature of abuse, and, therefore, cannot endure active measures. Depletion is badly borne; abstinence, even moderately practised, is often attended with unfavourable consequences. As evacuants are the best class of medicines for the sanguine, perhaps tonics are the safest for the nervous. Their relaxed fibre, their enervated mind, their turbid passions, their debilitated functions, their whole constitutional *status* is the result of weakness, and can only be removed by communicating strength. Every plan by which tone can be imparted to the body, and firmness to the mind, should be judiciously employed. Exercise properly conducted, amusements moderately enjoyed, temperance in rest, food, drink, and passions, tonics carefully administered, and, perhaps, sedatives and antispasmodics occasionally given, are the most promising means for accomplishing this object.

We have now brought to a close our observations upon *Temperaments*. We have examined their history—investigated their causes—ascertained the leading principles of their constitution; we have inquired into their connexion with mind—have defined the limits of their intimacy with matter—have shewn how they may depend upon both, and how original confirmation may be modified by accident; we have seen how they influence the system in a state of health, giving to one energy of function and vivacity of thought, to another sensibility of body and volatility of mind, and to a third corporeal apathy, and metaphysical dulness; we have observed to what extent they favour the establishment, the character, and the progress of disease; in this system discountenancing its attack but encouraging its advancement, in that hurrying forward action to a crisis, and in another obscuring every symptom, and resisting every remedy; and lastly, the indications to treatment which they hold out have been examined. Did space permit, a subject so extensive might be more extensively pursued, but we believe the assigned limits have been already overstepped, and it only remains for us in conclusion to observe, that since temperaments stand very much at the mercy of circumstance, whether they be favourable to health or to disease, to happiness or to misery, to mental cultivation or to moral wretchedness, and since the nature and influence of these circumstances are very much under our control, the advice of Pythagoras, with respect to habits, may be applied as forcibly to temperaments; *Maximum vitæ genus eligite, nam consuetudo faciet jucundissimum.*

